# Etymologia: Diphtheria

**DOI:** 10.3201/eid1911.ET1911

**Published:** 2013-11

**Keywords:** etymologia, diphtheria, Corynebacterium diphtheriae, bacteria, pseudomembrane, throat, Pierre Bretonneau

## Diphtheria [dif-thēr**′**e-ə]

From the Greek *diphthera* (leather), diphtheria is named for the tough pseudomembrane that forms in the patient’s throat. One of the earliest accounts of what may have been symptoms of diphtheria is found in Hippocrates work *Epidemics III*, written 2,500 years ago. Reports of epidemics of “throat distemper” began to appear in the 1500s, but before the 19th century, diphtheria was known around the world by many different names, such as Syrian ulcer, membranous angina, malignant croup, and Boulogne sore throat. In 1821, French physician Pierre Bretonneau described diphtheria’s unique clinical characteristics during an epidemic in southern France, when he named it *diphtérite* after the leathery texture of the pseudomembrane.
